# Risk factors and their interaction on chronic kidney disease: A multi-centre case control study in Taiwan

**DOI:** 10.1186/s12882-015-0065-x

**Published:** 2015-06-16

**Authors:** Sui-Lung Su, Chin Lin, SenYeong Kao, Chia-Chao Wu, Kuo-Cheng Lu, Ching-Huang Lai, Hsin-Yi Yang, Yu-Lung Chiu, Jin-Shuen Chen, Fung-Chang Sung, Ying-Chin Ko, Chien-Te Lee, Yu Yang, Chih-Wei Yang, Shang-Jyh Hwang, Ming-Cheng Wang, Yung-Ho Hsu, Mei-Yi Wu, Yu-Mei Hsueh, Hung-Yi Chiou, Yuh-Feng Lin

**Affiliations:** School of Public Health, National Defense Medical Center, Taipei, Taiwan; Graduate Institute of Life Sciences, National Defense Medical Center, Taipei, Taiwan; Division of Nephrology, Department of Medicine, Tri-Service General Hospital, National Defense Medical Center, Taipei, Taiwan; Division of Nephrology, Department of Medicine, Cardinal Tien Hospital, School of Medicine, Fu Jen Catholic University, New Taipei City, Taiwan; School of Public Health, Graduate Institute of Clinical Medical Science, China Medical University, Taichung, Taiwan; Division of Nephrology, Kaohsiung Chang Gung Memorial Hospital, Chang Gung Medical University, Kaohsiung, Taiwan; The Division of Nephrology, Changhua Christian Hospital, Changhua, Taiwan; School of Medicine, Chang Gung University, Taoyuan, Taiwan; Division of Nephrology, Department of Medicine, Kaohsiung Medical University Hospital, Kaohsiung, Taiwan; Department of internal Medicine, Cheng Kung University Medicial Center, Tainan, Taiwan; Division of Nephrology, Department of Medicine, Shuang Ho Hospital, Graduate Institute of Clinical Medicine, Taipei Medical University, No. 250, Wuxing St., Xinyi District, Taipei, 110 Taiwan; School of Public Health, Taipei Medical University, No. 250, Wuxing St., Xinyi District, Taipei, 110 Taiwan

**Keywords:** Chronic kidney disease, Hypertension, Anaemia, Hyperlipidaemia, Interaction

## Abstract

**Background:**

Chronic kidney disease (CKD) is highly prevalent in Taiwan. More than two-thirds of end-stage renal disease is associated with diabetes mellitus (DM) or hypertension (HTN). Therefore, the formulation of a special preventative policy of CKD in these patients is essential. This study surveyed 14 traditional risk factors and identified their effects on CKD in patients with HTN/DM and compared these with their effects in the general population.

**Methods:**

This study included 5328 cases and 5135 controls in the CKD/HTN/DM outpatient and health centres of 10 hospitals from 2008 to 2010. Fourteen common effect factors were surveyed (four demographic, five disease and five lifestyle), and their effects on CKD were tested. Significance tests were adjusted by the Bonferroni method. Results of the stratified analyses in the variables were presented with significant heterogeneity between patients with different comorbidities.

**Results:**

Male, ageing, low income, hyperuricemia and lack of exercise habits were risk factors for CKD, and their effects in people with different comorbidities were identical. Anaemia was a risk factor, and there was an additive effect between anaemia and HTN on CKD. Patients with anaemia had a higher risk when associated with HTN [odds ratio (OR) = 6.75, 95 % confidence limit (95 % CI) 4.76–9.68] but had a smaller effect in people without HTN (OR 2.83, 95 % CI 2.16–3.67). The association between hyperlipidaemia-related factors and CKD was also moderated by HTN. It was a significant risk factor in people without HTN (OR = 1.67, 95 % CI 1.38–2.01) but not in patients with HTN (OR =1.03, 95 % CI 0.89–1.19). Hepatitis B, hepatitis C, betel nut chewing, smoking, alcohol intake and groundwater use were not associated with CKD in multivariate analysis.

**Conclusions:**

We considered that patients with HTN and anaemia were a high CKD risk population. Physicians with anaemic patients in outpatient clinics need to recognise that patients who also have HTN might be latent CKD cases.

**Electronic supplementary material:**

The online version of this article (doi:10.1186/s12882-015-0065-x) contains supplementary material, which is available to authorized users.

## Background

Chronic kidney disease (CKD) is an important public health issue because these patients have an increased risk of end-stage renal disease (ESRD). Taiwan has a high prevalence of CKD [1] and ESRD [2]. These patients are at increased risk for cardiovascular events and progression to kidney failure [3]. The benefits of screening at-risk populations and estimating progression of CKD are well established [4]. A previous study has shown that screening people with hypertension (HTN), diabetes mellitus (DM) or age >55 years is the most effective strategy to detect patients with CKD [5]. Therefore, planning a specific population screening/prevention strategy for people with HTN or DM is a major public health challenge. To our knowledge, there is no systematic evidence at present to confirm that a screening/prevention strategy for the general population would apply to high risk groups.

CKD is a complex disease that has complex aetiologies, but the effects of these factors are mild. The traditional factors that have an effect on CKD are primarily divided into three parts: demographic characteristics (gender [6], age [7], obesity [8] and social economic [9]), comorbidity [hepatitis B (HB) [10], hepatitis C (HC) [11], hyperuricemia [7], anaemia [12] and hyperlipidaemia [7]] and lifestyles (smoking status [13], alcohol intake [14], betel nut chewing [15], exercise habits [16, 17] and groundwater use [18]). Foregoing factors have been extensively investigated and some studies have investigated their effects in populations. However, these reported effects are inconsistent in different populations. For example, obesity had a significant effect in the general population [8] but not in patients with DM [19]. This suggests that the effects of obesity on CKD may be associated with DM. In addition, several studies have investigated the interaction between some of these factors and HTN/DM on CKD. Other studies have reported an interaction between HTN and smoking [20] and between DM and hyperuricemia [21] on renal outcomes. As a result of these various reports, we suspected that the effect of each factor on CKD may be different in healthy populations and in patients with DM/HTN.

Studies have shown evidence that the effects of some factors on CKD may depend on the presence of DM or HTN, which means that some risk factors may or may not be important in patients with HTN/CKD. This information may be important for clinical decision making for CKD patients with HTN/CKD. However, to our knowledge, no study has systematically investigated the potential factors which may have a DM- or HTN-dependent effect on CKD. Therefore, the aim of this study was to investigate different targets for screening/prevention strategies between healthy people and patients with HTN or DM that may aid in planning most effective prevention strategies for patients with DM and HTN.

## Methods

### Population and definition

A multi-centre project in January 2008 to July 2010 was conducted to survey the risk factors for CKD in Taiwan. Fourteen hospitals equally participated in the program. Before starting the study, appropriate sample size estimation was calculated using OpenEpi (http://www.openepi.com/SampleSize/SSCC.htm). The settings used were: a two-sided test with a power of (1 − β) = 0.95 at a significance level of α = 0.05/14, the ratio of controls to cases = 1, the hypothetical proportion of controls with exposure = 5 and the least extreme odds ratio (OR) to be detected = 1.5. Based on these settings, the study sample size required was at least 9104 subjects.

**Fig. 1:**
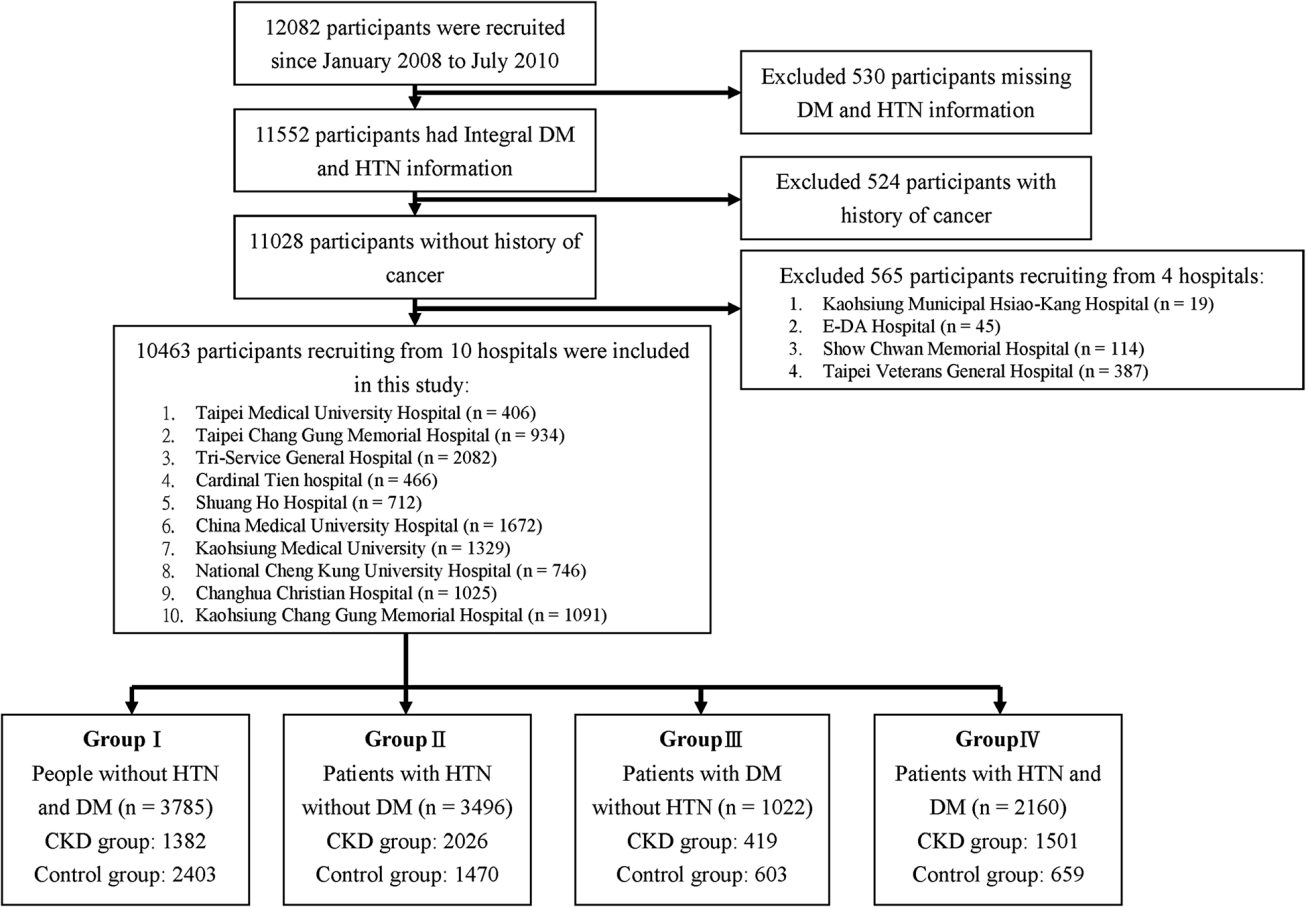
Recruitment Process Flow Chart HTN: hypertension; DM: diabetes mellitus; CKD: chronic kidney disease;n: number of participants were meeting for specific group. We recruited participants without CKD from the HTN/DM outpatient and health centres in each hospital as a control group. The CKD cases were recruited from both the nephrology and HTN/DM outpatient clinics in each hospital and from health centres

To identify the specific risk factors in patients with HTN/DM and to maintain the statistical power of this study, a higher number of patients with HTN/DM were required. Based on this, we also recruited participants without CKD from HTN/DM outpatient and health centres from each hospital as the control group. The CKD cases were recruited from nephrology and HTN/DM outpatient clinics in each hospital and from health centres and were classified as the case group. Finally, a total of 12,082 participants older than 18 years were recruited consecutively in this project (Fig. 1).

The study included 11,552 (95.6 %) participants with no missing DM and HTN status information. Exclusions included 524 participants who had cancer and 565 from four hospitals because of insufficient recruitment numbers (<150 participants from three hospitals and one had only 75 healthy controls). Finally, a total of 10,463 (5218 male and 5245 female) participants (median age 57.0 years) from 10 different hospitals were included in the study.

CKD was defined as an estimated glomerular filtration rate (eGFR) of <60 mL/min/1.73 m^2^ or with proteinuria. The eGFR was calculated with the equation eGFR (mL/min/1.73 m^2^) = 186 × Cr^−1.154^ × age^−0.203^ × 0.742 (if female) [22]. A total of 5328 participants that met the above criteria were included in the cases group and 5135 others in the control group. The different stages of CKD and non-CKD was defined as follows: CKD Stage 1 eGFR ≥ 90 with albumin excretion ≥150 mg and microhematuria and/or renal parenchymal disease, CKD Stage 2 eGFR 60.0–89.9 with above mentioned criteria, CKD Stage 3 eGFR 30.0–59.9, CKD Stage 4 eGFR 15.0–29.9, CKD Stage 5 eGFR ˂15, and non-CKD eGFR ≥60 without above mentioned criteria.

The definition of diabetes and HTN was based on physician diagnoses using a fasting glucose level of >126 mg/dL [23] and a systolic/diastolic blood pressure level of >130/80 mmHg [24], respectively. The study participants were placed into four groups according to their disease status: Group I consisted of 3785 participants without both DM and HTN (1382 cases and 2403 controls), Group II consisted of 3,496 participants with HTN and without DM (2026 cases and 1470 controls), Group III consisted of 1022 participants with DM and without HTN (419 cases and 603 controls) and Group IV consisted of 2160 participants with both DM and HTN (1501 cases and 659 controls).

### Risk factor assessment and definition

Demographic characteristics (gender, age, obesity and socioeconomic), history of disease (HB, HC, hyperuricemia, anaemia and hyperlipidaemia) and lifestyle (smoking status, alcohol intake, betel nut chewing, exercise habits and use of groundwater status) were the possible risk factors that were investigated for their individual effects and for the different effects on CKD in the four study groups. A detailed medical history, anthropometric measurements, laboratory analyses, and a health appraisal questionnaire eliciting demographic, socioeconomic and behavioural risk factors were conducted through face-to-face interviews with each participant by well-trained investigators at the initial visit. Written informed consent was obtained from all study participants.

Gender and age were assessed based on self-reporting. Body mass index (BMI) was calculated as weight/height^2^ (kg/m^2^) and participants whose BMI was >27 were classified as obese. The socioeconomic status was based on income and individuals were divided into three groups: low income was defined as less than 20,000 New Taiwan Dollars (NTD) per month, median income was between 20,000 and 60,000 NTD per month and high income was more than 60,000 NTD per month (1 US dollar = 30 NTD).

Participants were recorded with a history of disease if there was an affirmative answer to having ever been diagnosed by a doctor with HB, HC, hyperuricemia, anaemia or hyperlipidaemia. The diagnosis of anaemia used in Taiwan is defined by the WHO as haemoglobin <12 g/dL in women and <13 g/dL in men [25].

Smoking status (current, former or never) was ascertained at the time of enrolment. Individuals who had not smoked more than 100 cigarettes in their lifetime were classified as never-smokers, based on common conventions in epidemiology research [26]. Others were grouped together in the smokers group.

Alcohol intake was defined as total alcohol consumption in grams per day. We calculated this from questions about different liquors, frequencies and concentrations.

A previous study reported that moderate alcohol consumption (10–20 g/day) is associated with favourable levels of several cardiovascular risk factors [27]. Therefore, individuals who had intakes of >20 g/day that continued for more than 1 month were classified in the ever-drink group, and others were classified in the never-drink group.

Betel nut chewing and exercise habits were based on a self-report questionnaire. If the participants gave a positive answer to having ever had habits of betel nut chewing or exercise, they were classified in the ever-group and the others in the never-group. Individuals who participated in physical activity fewer than three times/week were classified in the never-group [26].

Using groundwater was ascertained by the participants as having and using stable groundwater. Individuals who used groundwater for a period of more than 1 year were classified as the ever-used group and others were classified as the never-used group.

### Ethics statement

The study was reviewed and approved by the institutional ethical committee of Taipei Medical University Hospital (201204035), Taipei Chang Gung Memorial Hospital (97-2187B), Tri-Service General Hospital (100-05-197), Cardinal Tien Hospital (201204035), Shuang Ho Hospital (201204035), China Medical University Hospital [DMR101-IRB2-273 (CR-1)], Kaohsiung Medical University Chung-Ho Memorial Hospital (KMUH-IRB-20120019), National Cheng Kung University Hospital (ER-101-117), Changhua Christian Hospital (120405) and Kaohsiung Chang Gung Memorial Hospital (101-1096B). After a complete explanation of the study, written informed consent was obtained from all participants. All clinical and biological samples were collected and DNA was genotyped following patient consent.

### Statistical analysis

The total cohort, including participants from different sources (HTN/DM outpatient and health centres) may have distorted the proportion of cases/controls with HTN/DM, and this could have resulted in the wrong risk assessments of HTN/DM and some of the factors relating to HTN/DM. Therefore, this study did not investigate the risk of HTN/DM on CKD, because the exposure rate of HTN/DM may be biased. The HTN/DM cases were stratified in all analyses in order to investigate the interactions between HTN/DM and the other factors, and the stratified analyses could help reduce the confounding effect.

The significance tests were adjusted by the Bonferroni method and a *p*-value of <0.05/14 = 0.0036 was considered significant for avoiding the error of multiple testing. Statistical analyses were conducted with R 3.0.1 software with the ‘lme4’, ‘meta’ and ‘metafor’ package. The dependent variable for the analyses was patients with CKD. Categorical and continuous variables were presented as the number (proportion) and mean ± standard deviation (SD). The post powers were calculated based on G*Power 3.1.7 [28].

To test the effect of each factor and to control the hospital-clustering effect (Simpson paradox), all factors were tested using hierarchical generalised linear models in the four groups (Groups I–IV). The OR and 95 % confidence interval (95 % CI) were presented for the effects of risk factors on CKD. All variables were adjusted in multivariate analyses regardless of whether they were significant in the univariate analyses. Meta-analysis was used to quantify the difference between the coefficients of a specific variable in the different groups. The I^2^ test for estimating the heterogeneity between the four groups and the Cochrane Q test were used. An I^2^ > 75 % represented a high heterogeneity and implied that the interaction between that variable and HTN/DM may exist [29]. The τ^2^ statistic was estimated by the restricted maximum-likelihood estimator method, and a random-effects model based on the Mantel–Haenszel method was applied. When the heterogeneity of the coefficients of a specific variable was significant in the multivariate analyses, we presented the results of the stratified analyses and attempted to find the cause of heterogeneity (DM or HTN). Moreover, the results of the pooled analyses were considered more reliable in study populations. The change of τ^2^ statistic that was estimated was used for assessment for the cause of heterogeneity.

## Results

Table 1 shows the proportion of cases and controls with exposure to all variables. In the post power assessment, the powers of HC (39.6 %), anaemia (92.1 %) and betel nut chewing (82.2 %) were <95 %, and the powers of the other variables were >95 %.

**Table 1:** Overall proportion of cases and controls with exposure

		CKD	non-CKD	Post power
Gender	Female	2302 (43.2 %)	2943 (57.3 %)	>99.9 %
	Male	3026 (56.8 %)	867 (42.7 %)	
Age		59.8 ± 15.2	54.0 ± 15.1	>99.9 %
Obesity (151 missing)	Normal	4634 (88.7 %)	4650 (91.4 %)	>99.9 %
	Abnormal	592 (11.3 %)	436 (8.6 %)	
Income (61 missing)	Low	3056 (57.6 %)	2039 (40.0 %)	
	Median	1416 (26.7 %)	1791 (35.1 %)	>99.9 %
	High	832 (15.7 %)	1268 (24.9 %)	>99.9 %
HB (1 missing)	Normal	5027 (94.4 %)	5064 (93.9 %)	99.4 %
	Abnormal	300 (5.6 %)	71 (6.1 %)	
HC* (2 missing)	Normal	5228 (98.2 %)	5064 (98.6 %)	39.6 %
	Abnormal	98 (1.8 %)	71 (1.4 %)	
Hyperuricaemia (2 missing)	Normal	4036 (75.8 %)	4852 (94.5 %)	98.7 %
	Abnormal	1291 (24.2 %)	282 (5.5 %)	
Anaemia* (2 missing)	Normal	4463 (83.8 %)	4938 (96.2 %)	92.1 %
	Abnormal	863(16.2 %)	197 (3.8 %)	
Hyperlipidaemia (1 missing)	Normal	3832 (71.9 %)	4196 (81.7 %)	>99.9 %
	Abnormal	1495 (28.1 %)	939 (18.3 %)	
Smoking status (338 missing)	Never	3909 (76.0 %)	4126 (82.9 %)	>99.9 %
	Ever	1237 (24.0 %)	853 (17.1 %)	
Alcohol intake (382 missing)	Never	4361 (85.0 %)	4340 (87.6 %)	>99.9 %
	Ever	767 (15.0 %)	613 (12.4 %)	
Betel nut chewing* (456 missing)	Never	4876 (95.7 %)	4758 (96.9 %)	82.2 %
	Ever	222 (4.3 %)	151 (3.1 %)	
Exercise habits (173 missing)	Never	1862 (35.8 %)	1511 (29.7 %)	>99.9 %
	Ever	3338 (64.2 %)	3579 (70.3 %)	
Groundwater using (17 missing)	Never	5029 (94.5 %)	4890 (95.4 %)	96.6 %
	Ever	293 (5.5 %)	234 (4.6 %)	

CKD: patients with CKD; non-CKD: patients without CKD; HB: hepatitis B; HC: hepatitis C

§: Post power (1-β) estimate based on G*power [28]

Boldface & *: the powers of each variable were less than 95 %, and they were defined as lacking in power

The characteristics of the case and control groups in Groups I–IV are shown in Table 2, and the hierarchical generalised linear models were used to test the difference of proportion between cases and controls by controlling the hospital-clustering effects in each group (stratified analyses). Detailed information is shown in the supplementary file (univariate analyses Additional file 1: Table S1 and multivariate analyses Additional file 1: Table S2). Based on Additional file 1: Table S1 and Additional file 1: Table S2, Table 3 shows the pooled results of all variables.

**Table 2:** Characteristics of subjects by stratification for HTN and DM

		Group I		Group II		Group III		Group IV	
		CKD	non-CKD	CKD	non-CKD	CKD	non-CKD	CKD	non-CKD
Gender	Female	670 (48.5 %)	1536 (63.9 %)	819 (40.4 %)	752 (51.2 %)	172 (41.1 %)	304 (50.4 %)	641 (42.7 %)	351 (53.3 %)
	Male	712 (51.5 %)	867 (36.1 %)	1207 (59.6 %)	718 (48.8 %)	247 (58.9 %)	299 (49.6 %)	860 (57.3 %)	308 (46.7 %)
Age		53.0 ± 17.1	47.3 ± 15.2	61.3 ± 15.1	60.5 ± 13.0	61.7 ± 12.1	57.6 ± 12.1	63.5 ± 11.8	60.8 ± 10.8
Obesity (151 missing)	Normal	1278 (93.6 %)	2234 (94.0 %)	1787 (89.8 %)	1311 (90.1 %)	356 (86.2 %)	538 (89.8 %)	1213 (83.3 %)	567 (86.6 %)
	Abnormal	87 (6.4 %)	143 (6.0 %)	204 (10.2 %)	144 (9.9 %)	57 (13.8 %)	61 (10.2 %)	244 (16.7 %)	88 (13.4 %)
Income (61 missing)	Low	648 (47.3 %)	677 (28.3 %)	1153 (57.0 %)	736 (50.3 %)	265 (63.4 %)	260 (44.0 %)	990 (66.4 %)	366 (56.2 %)
	Median	434 (31.7 %)	971 (40.6 %)	554 (27.4 %)	418 (28.6 %)	97 (23.2 %)	213 (36.0 %)	331 (22.2 %)	189 (29.0 %)
	High	289 (21.1 %)	746 (31.2 %)	316 (15.6 %)	308 (21.1 %)	56 (13.4 %)	118 (20.0 %)	171 (11.5 %)	96 (14.7 %)
HB (1 missing)	Normal	1273 (92.1 %)	2222 (92.5 %)	1905 (94.0 %)	1399 (95.2 %)	402 (95.9 %)	574 (95.2 %)	1447 (96.5 %)	629 (95.4 %)
	Abnormal	109 (7.9 %)	181 (7.5 %)	121 (6.0 %)	71 (4.8 %)	17 (4.1 %)	29 (4.8 %)	53 (3.5 %)	30 (4.6 %)
HC (2 missing)	Normal	1358 (98.3 %)	2377 (98.9 %)	1984 (97.9 %)	1446 (98.4 %)	412 (98.3 %)	593 (98.3 %)	1474 (98.3 %)	648 (98.3 %)
	Abnormal	23 (1.7 %)	26 (1.1 %)	42 (2.1 %)	24 (1.6 %)	7 (1.7 %)	10 (1.7 %)	26 (1.7 %)	11 (1.7 %)
Hyperuricemia (2 missing)	Normal	1185 (85.7 %)	2335 (97.2 %)	1398 (69.0 %)	1336 (90.9 %)	367 (87.6 %)	572 (94.9 %)	1086 (72.4 %)	609 (92.4 %)
	Abnormal	197 (14.3 %)	68 (2.8 %)	628 (31.0 %)	133 (9.1 %)	52 (12.4 %)	31 (5.1 %)	414 (27.6 %)	50 (7.6 %)
Anaemia (2 missing)	Normal	1217 (88.1 %)	2285 (95.1 %)	1674 (82.6 %)	1431 (97.3 %)	383 (91.4 %)	588 (97.5 %)	1189 (79.3 %)	634 (96.2 %)
	Abnormal	165 (11.9 %)	118 (4.9 %)	352 (17.4 %)	39 (2.7 %)	36 (8.6 %)	15 (2.5 %)	310 (20.7 %)	25 (3.8 %)
Hyperlipidaemia (1 missing)	Normal	1144 (82.8 %)	2184 (90.9 %)	1478 (73.0 %)	1123 (76.4 %)	308 (73.5 %)	460 (76.3 %)	902 (60.1 %)	429 (65.1 %)
	Abnormal	238 (17.2 %)	219 (9.1 %)	548 (27.0 %)	347 (23.6 %)	111 (26.5 %)	143 (23.7 %)	598 (39.9 %)	230 (34.9 %)
Smoking status (338 missing)	Never	1088 (80.5 %)	2041 (87.6 %)	1496 (76.6 %)	1148 (80.8 %)	288 (71.1 %)	438 (74.9 %)	1037 (72.2 %)	499 (77.7 %)
	Ever	263 (19.5 %)	290 (12.4 %)	457 (23.4 %)	273 (19.2 %)	117 (28.9 %)	147 (25.1 %)	400 (27.8 %)	143 (22.3 %)
Alcohol intake (382 missing)	Never	1203 (89.0 %)	2109 (91.1 %)	1658 (85.2 %)	1209 (85.4 %)	338 (83.9 %)	480 (82.2 %)	1162 (81.4 %)	542 (85.0 %)
	Ever	148 (11.0 %)	207 (8.9 %)	289 (14.8 %)	206 (14.6 %)	65 (16.1 %)	104 (17.8 %)	265 (18.6 %)	96 (15.0 %)
Betel nut chewing (456 missing)	Never	1296 (96.7 %)	2257 (98.5 %)	1870 (96.6 %)	1360 (97.0 %)	374 (93.0 %)	539 (92.8 %)	1336 (94.1 %)	602 (95.0 %)
	Ever	44 (3.3 %)	35 (1.5 %)	66 (3.4 %)	42 (3.0 %)	28 (7.0 %)	42 (7.2 %)	84 (5.9 %)	32 (5.0 %)
Exercise habits (173 missing)	Never	474 (34.8 %)	755 (31.7 %)	691 (35.0 %)	389 (26.7 %)	138 (33.5 %)	173 (28.9 %)	559 (38.6 %)	194 (29.6 %)
	Ever	887 (65.2 %)	1624 (68.3 %)	1286 (65.0 %)	1068 (73.3 %)	274 (66.5 %)	426 (71.1 %)	891 (61.4 %)	461 (70.4 %)
Groundwater using (17 missing)	Never	1312 (95.2 %)	2334 (97.3 %)	1904 (94.0 %)	1349 (92.0 %)	394 (94.0 %)	574 (95.5 %)	1419 (94.7 %)	633 (96.2 %)
	Ever	66 (4.8 %)	65 (2.7 %)	122 (6.0 %)	117 (8.0 %)	25 (6.0 %)	27 (4.5 %)	80 (5.3 %)	25 (3.8 %)

Group I: participants without DM and HTN; Group II: participants with HTN without DM; Group III: participants with DM without HTN; Group IV: participants with DM and HTN

CKD: patients with CKD; non-CKD: patients without CKD; HTN: hypertension; DM: diabetes mellitus; HB: hepatitis B; HC: hepatitis C

**Table 3:** Pooled data showing effect of each risk factor on CKD and heterogeneity between the four groups§

	Univariable analyses			Multivariable analyses		
	OR (95 % CI)	I^2^	Q test	OR (95 % CI)	I^2^	Q test
Gender (Male versus Female)	1.65 (1.49 to 1.82)*	25.5 %	0.296	1.61 (1.45 to 1.78)*	0.0 %	0.934
Age (per 10 years)	1.19 (1.09 to 1.29)*£	85.7 %*	<0.001*	1.44 (1.13 to 1.83)*	24.1 %	0.323
Obesity (Abnormal versus Normal)	1.14 (0.93 to 1.40)	43.9 %	0.163	1.07 (0.86 to 1.33)	39.0 %	0.204
Income (Median versus Low)	0.57 (0.45 to 0.72)*£	80.8 %*	<0.001*	0.58 (0.47 to 0.73)*	71.9 %	0.011
(High versus Low)	0.50 (0.38 to 0.67)*£	82.3 %*	<0.001*	0.55 (0.43 to 0.70)*	65.0 %	0.022
HB (Abnormal versus Normal)	1.08 (0.88 to 1.33)	21.6 %	0.263	1.25 (1.03 to 1.52)	0.0 %	0.523
HC (Abnormal versus Normal)	1.29 (0.93 to 1.79)	0.0 %	0.707	1.22 (0.85 to 1.74)	0.0 %	0.778
Hyperuricaemia (Abnormal versus Normal)	4.56 (3.96 to 5.26)*	0.0 %	0.213	3.63 (3.11 to 4.24)*	0.0 %	0.806
Anaemia (Abnormal versus Normal)	4.89 (2.76 to 8.66)*£	88.5 %*	<0.001*	4.64 (2.81 to 7.65)*£	82.2 %*	<0.001*
Hyperlipidaemia (Abnormal versus Normal)	1.48 (1.17 to 1.88)*£	80.5 %*	0.001	1.28 (0.97 to 1.67)£	81.3 %*	0.001*
Smoking status (Abnormal versus Normal)	1.45 (1.28 to 1.64)*	26.6 %	0.305	1.17 (1.02 to 1.34)	0.0 %	0.559
Alcohol intake (Abnormal versus Normal)	1.15 (1.00 to 1.33)	22.3 %	0.248	0.83 (0.71 to 0.97)	0.0 %	0.701
Betel nut chewing (Abnormal versus Normal)	1.35 (1.03 to 1.77)	29.0 %	0.233	1.10 (0.85 to 1.44)	0.0 %	0.836
Exercise habits (Abnormal versus Normal)	0.74 (0.65 to 0.85)*	50.9 %	0.106	0.71 (0.64 to 0.79)*	0.0 %	0.859
Groundwater using (Abnormal versus Normal)	1.44 (1.13 to 1.83)	24.1 %	0.323	1.29 (1.04 to 1.61)	0.0 %	0.534

§: The four groups were Group I (participants without DM and HTN), Group II (participants with HTN without DM), Group III (participants with DM without HTN) and Group IV (participants with DM and HTN)

HB: hepatitis B; HC: hepatitis C

OR: pooled odds ratio for variation groups compared with reference groups on CKD; 95 % CI: 95 % confidence interval of OR

I^2^: heterogeneity between four groups in each variable; Q test: the significant test of I^2^ using Cochrane Q test

Boldface & *: significance after Bonferroni adjustment: p value <0.05/14 = 0.0036

£: The pooled results were unreliable because the difference between coefficients in four group were significant. Please refer to the results of stratified analyses in Supplementary File (univariable analyses: Additional file 1: Table S1; multivariable analyses: Additional file 1: Table S2)

Male (*p* <0.001), hyperuricemia (*p* <0.001), smoking (*p* <0.001), lack of exercise habits (*p* <0.001) and groundwater use (*p* = 0.003) were significant risk factors for CKD in the univariate analyses, and their heterogeneities were not significant. In the multivariate analyses, male (*p* <0.001), hyperuricemia (*p* <0.001) and lack of exercise habits (*p* <0.001) were significant and their heterogeneities were not significant. However, smoking (*p* = 0.026) and groundwater use (*p* = 0.022) were not significant after adjustment by the Bonferroni method. The pooled ORs of gender, hyperuricemia and exercise habits were 1.61 (95 % CI = 1.45–1.78, *p* <0.001), 3.63 (95 % CI = 3.11–4.24, *p* <0.001) and 0.71 (95 % CI = 0.64–0.79, *p* <0.001). Age and income may relate to CKD, but they had high heterogeneity between the four groups (I^2^ of age 85.7 %, I^2^ of income 80.8 % and 82.3 %). This implies that the pooled ORs of these variables may be unreliable. However, the heterogeneities were significantly reduced after the adjusted demographic characteristics, the history of disease and lifestyle. The pooled ORs per 10 years of age was 1.44 (95 % CI = 1.13–1.83, *p* = 0.003) and of income for Median vs. Low was 0.58 (95 % CI = 0.47–0.73 *p* <0.001) and for High vs. Low was 0.55 (95 % CI = 0.43–0.70, *p* <0.001), which were all statistically significant.

The heterogeneities of obesity, HB, HC, alcohol intake and betel nut chewing were not significant in the univariate/multivariate analyses, and their pooled ORs were not significant. Based on the power of this study, obesity, HB and alcohol intake may not be associated with CKD (unless their true ORs were very small), but the power of the significance test in HC and betel nut chewing may be insufficient.

**Fig. 2:**
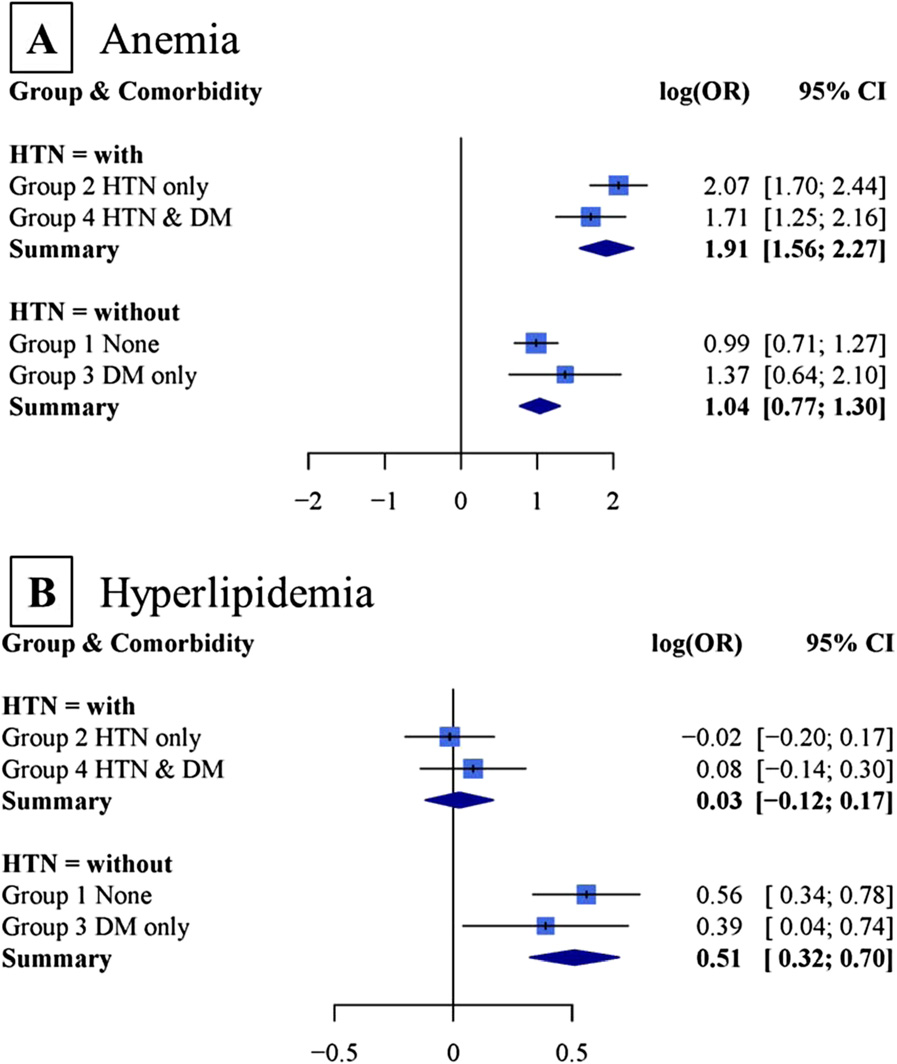
Stratified analyses for variables with significant heterogeneity between the four groups using multivariable analyses. **(a)** shows coefficients of anaemia and **(b)** shows coefficients of hyperlipidaemia using multivariate analyses and their heterogeneity caused by HTN in the different groups. Amount of heterogeneity accounted for by HTN was 93.71 %/100 %. The odds ratios and 95 % CI of anaemia in patients with or without HTN were 6.75 (4.76–9.68) and 2.83 (2.16–3.67), respectively. The odds ratios (95 % CI) of hyperlipidaemia in patients with or without HTN were 1.03 (0.89–1.19) and 1.67 (1.38–2.01), respectively HTN: hypertension; DM: diabetes mellitus; log (OR): natural logarithm of the odds ratio; 95 % CI: 95 % confidence interval of log (OR)

The results of anaemia and hyperlipidaemia were interesting. We found that there was high and significant heterogeneity among the four groups. The I^2^ of anaemia was 88.5 % for univariate and 82.2 % for multivariate analyses, and the I^2^ of hyperlipidaemia was 80.5 % for univariate and 81.3 % for multivariate analyses. These results imply that the pooled ORs may be unreliable. Fig. 2 shows further analyses that indicate the causes of heterogeneity for anaemia (93.7 %) and hyperlipidaemia (100 %) may be due to HTN. After the recombined analyses, the ORs of anaemia and hyperlipidaemia were 6.75 (95 % CI = 4.76–9.68, *p* <0.001) and 1.03 (95 % CI = 0.89–1.19, *p* = 0.695), respectively, in patients with HTN, and the ORs of anaemia and hyperlipidaemia were 2.83 (95 % CI = 2.16–3.67, *p* <0.001) and 1.67 (95 % CI = 1.38–2.01, *p* <0.001), respectively, in people without HTN. These results represent an additive interaction effect of anaemia and HTN on CKD, and an antagonistic interaction effect of hyperlipidaemia and HTN on CKD.

## Discussion

The results of this study found that male, ageing, low income, hyperuricemia and lack of exercise habits were risk factors for CKD. The effects of anaemia and hyperlipidaemia on CKD in patients with or without HTN were different. We also found that HB, HC, smoking, alcohol intake, betel nut chewing and groundwater use may not be associated with CKD. Among males, ageing and lack of exercise habits were the traditional risk factors for CKD [7, 6, 17, 16]. In our study, we also observed a significant association between these risk factors and CKD, and we did not find high heterogeneity among the four groups. The discussion that follows divides the associations between other variables and CKD into three parts: socioeconomic status-related factors, possible reasons for the high prevalence of ESRD in Taiwan and the interactive effects between anaemia/hyperlipidaemia and HTN on CKD.

Low income was an important predictive factor for CKD, and it was characteristic of people with a low socioeconomic status. Socioeconomic status may be related to many risk factors, such as second-hand tobacco smoke [30] and unhealthy diets [31]. In this study, we investigated some socioeconomic status-related factors (smoking status [32], alcohol intake [33], betel nut chewing [34] and groundwater use [18]), and we found that the association between smoking status/groundwater use and CKD may be related to income level. Their effects were significant before the adjustment of income but not after this adjustment.

The ORs of alcohol intake and betel nut chewing in the univariate and multivariate analyses also presented a similar phenomenon. Previous studies that had investigated the above factors did not adjust for socioeconomic status. Thus, they may have overrated their risk on CKD [14, 15, 18]. Based on the power of this study, further research is needed concerning the association between betel nut chewing and CKD. With smoking status, alcohol intake and groundwater use, these may have a smaller impact on CKD. Intervention for these small impact factors may not be effective, and therefore, we determined that smoking status, alcohol intake and betel nut chewing were not the best targets for CKD prevention.

The prevalence of HB [35] and HC [36] in Taiwan is higher than in most other countries, and Taiwan has the highest prevalence and third highest incidence of ESRD in the world [2]. Previous studies have reported the association between HC and CKD [11], so we suspected that the high prevalence of hepatitis might be the main reason for the high prevalence of ESRD. However, our study showed the nonsignificance of these factors. Therefore, we determined that HB and HC were not the main reason for the high prevalence and incidence of ESRD in Taiwan, but the power of HC may be insufficient and needs further research.

People with hyperuricemia have a higher risk of ESRD than those without it, and the association was found to be very high in this study. Despite the lack of detailed statistics worldwide, we believe that the prevalence of hyperuricemia in Taiwan may be higher than in many other countries [37, 38], and this may also play a key role in ESRD in Taiwan. However, our study might have overrated the risk of hyperuricemia in CKD. Previous studies presented a significant association between hyperuricemia and CKD, but the relative risks in these studies were <2 [7, 21]. Therefore, we determined that hyperuricemia might be associated with CKD, but the effect of intervention for hyperuricemia-related factors on CKD might not be very effective.

Anaemia is a likely complication in patients with CKD, but this is not a cause [39]. Therefore, it is not a good target for the prevention of CKD. However, this study was not only concerned with prevention strategies but also with screening strategies for CKD. Previous studies have demonstrated that awareness of CKD in patients is very low [40, 41]. Therefore, physicians might have to help them with monitoring the CKD. This study showed a strong association between anaemia and CKD in patients with HTN but not in the general population. This result helps to better pinpoint CKD high risk groups. Patients with both anaemia and HTN are a newly discovered high risk group for CKD, and physicians in outpatient clinics need to recognise that patients with anaemia accompanied with HTN might be latent CKD cases.

The risk effect of hyperlipidaemia on CKD was only found to be in the general population, but not in patients with HTN as reported in an earlier study [7]. Male subjects had a higher prevalence of HTN than females, and the hazard ratio of hyperlipidaemia on CKD in males was lower than in females [7]. Obesity was a hyperlipidaemia-related factor, and the risk effects of obesity presented similar results (Additional file 1: Table S1 and Additional file 1: Table S2). Although the results were not significant, we still found that the ORs in patients with HTN were lower than those in people without HTN. In addition, the heterogeneity of obesity was 39.0 % in the multivariate analysis, which implies that HTN might be a moderate factor in the association between obesity-related factors and CKD. It has been reported that renal lipid accumulation is nephrotoxic and could play a role in CKD [42], but we are unable to explain why the association between hyperlipidaemia and CKD in patients with HTN has a negative impact. Further research is required to understand the basic mechanism.

This study has two limitations. First, a cross-sectional study is not the way to distinguish whether correlations were causative or not, and time relationships were not confirmed in this study. Most of our discussion has focused on this issue, and we surmised that associations between some factors and CKD might not be causative. Although these may not help prevent CKD, they are useful for planning a screening strategy. Low awareness of CKD is an important issue, and a good outpatient screening strategy is urgently needed. Second, the risk factors assessment was based on a structured questionnaire rather than on the laboratory data. This may have caused some misclassification. However, Taiwan has good medical accessibility and most people can easily obtain medical resources and understand their disease status. In addition, our interviewers were highly trained, and we regularly held meetings for feedback from the interviewers. This ensured the quality of our research and reduced the possibility of misclassification.

## Conclusions

Gender, age, income, hyperuricemia, anaemia, hyperlipidaemia and exercise habits were good targets for planning a screening/prevention strategy for CKD in healthy populations and in patients with DM. In addition, the important evidence from this study confirmed that HB, smoking status, alcohol intake and groundwater use were not good targets for CKD prevention. They were not associated with CKD or only had a low impact on the condition. We believe that intervention for related factors might not be an efficient method based on the strong statistical power in this study. The associations between HC/betel nut chewing and CKD require further research because they were underpowered in this study. Finally, this study suggested that a specific CKD screening/prevention strategy for patients with DM might not be efficient without laboratory data analyses, and the strategy for the general population could be used in patients with DM. Furthermore, we determined that a screening/prevention strategy for CKD in patients with HTN might differ from that of a healthy population. Hyperlipidaemia-related factors might not be a good target for patients with HTN, and physicians need to recognise that patients with HTN in anaemia outpatient clinics might be potential CKD patients. In addition, there is a need for better care in patients with anaemia and HTN and timely intervention is required when there are signs of deterioration.

## Additional file

Additional file 1: Table S1. Effect of risk factors on CKD by hierarchical generalised linear models. Table S2: Effect of risk factors on CKD after adjusted demographic characteristics and history of disease and lifestyle.

## Abbreviations

BMI: Body mass index
CI: Confidence interval
CKD: Chronic kidney disease
NTD: New Taiwan dollars
DM: Diabetes mellitus
eGFR: Estimated glomerular filtration rate
ESRD: End-stage renal disease
HTN: Hypertension
HB: hepatitis B
HC: hepatitis C
OR: Odds ratio
SD: Standard deviation
