# Supplementary material for: Risk factors and their interaction on chronic kidney disease: A multi-centre case control study in Taiwan
Source: BMC Nephrol. 2015 Jun 16;16:83. doi: 10.1186/s12882-015-0065-x (PMC4469431; doi:10.1186/s12882-015-0065-x)
Supplement: Additional file 1: Table S1. — Effect of risk factors on CKD by hierarchical generalised linear models. Table S2: Effect of risk factors on CKD after adjusted demographic characteristics and history of disease and lifestyle. [file 12882_2015_65_MOESM1_ESM.doc]

**Supplementary File**

TABLE S1. Effect of risk factors on CKD by hierarchical generalized linear models.

|  | GroupⅠ |  | GroupⅡ |  | GroupⅢ |  | GroupⅣ |
| --- | --- | --- | --- | --- | --- | --- | --- |
|  | OR (95% CI) |  | OR (95% CI) |  | OR (95% CI) |  | OR (95% CI) |
| Gender (Male versus Female) | 1.83 (1.60 to 2.11)* |  | 1.62 (1.41 to 1.87)* |  | 1.51 (1.15 to 1.97)* |  | 1.50 (1.25 to 1.81)* |
| Age (per 10 years) £ | 1.24 (1.18 to 1.29)* |  | 1.06 (1.01 to 1.11)* |  | 1.29 (1.15 to 1.45)* |  | 1.21 (1.12 to 1.31)* |
| Obesity (Abnormal versus Normal) | 1.20 (0.90 to 1.60) |  | 0.90 (0.70 to 1.15) |  | 1.29 (0.81 to 2.06) |  | 1.34 (1.00 to 1.80) |
| Income (Median versus Low) £ | 0.46 (0.39 to 0.54)* |  | 0.76 (0.65 to 0.90)* |  | 0.49 (0.35 to 0.67)* |  | 0.58 (0.46 to 0.72)* |
| (High versus Low) £ | 0.37 (0.31 to 0.44)* |  | 0.65 (0.54 to 0.79)* |  | 0.43 (0.29 to 0.64)* |  | 0.61 (0.46 to 0.82)* |
| HB (Abnormal versus Normal) | 1.07 (0.83 to 1.38) |  | 1.36 (1.00 to 1.85) |  | 0.75 (0.39 to 1.45) |  | 0.88 (0.55 to 1.40) |
| HC (Abnormal versus Normal) | 1.56 (0.87 to 2.80) |  | 1.37 (0.81 to 2.32) |  | 0.85 (0.30 to 2.35) |  | 1.06 (0.52 to 2.18) |
| Hyperuricaemia (Abnormal versus Normal) | 5.35 (4.00 to 7.16)* |  | 4.50 (3.66 to 5.53)* |  | 2.91 (1.78 to 4.75)* |  | 4.72 (3.44 to 6.47)* |
| Anaemia (Abnormal versus Normal) £ | 2.88 (2.23 to 3.72)* |  | 9.23 (6.50 to 13.09)* |  | 3.11 (1.62 to 5.96)* |  | 6.58 (4.30 to 10.06)* |
| Hyperlipidaemia (Abnormal versus Normal) £ | 2.08 (1.70 to 2.55)* |  | 1.25 (1.06 to 1.47) |  | 1.41 (1.03 to 1.93) |  | 1.31 (1.07 to 1.60) |
| Smoking status (Abnormal versus Normal) | 1.68 (1.39 to 2.03)* |  | 1.33 (1.12 to 1.58)* |  | 1.37 (1.01 to 1.85) |  | 1.39 (1.11 to 1.74) |
| Alcohol intake (Abnormal versus Normal) | 1.25 (0.99 to 1.58) |  | 1.05 (0.86 to 1.29) |  | 0.93 (0.65 to 1.33) |  | 1.36 (1.05 to 1.76) |
| Betel nut chewing (Abnormal versus Normal) | 2.02 (1.26 to 3.22)* |  | 1.11 (0.74 to 1.67) |  | 1.10 (0.65 to 1.87) |  | 1.35 (0.88 to 2.07) |
| Exercise habits (Abnormal versus Normal) | 0.86 (0.74 to 0.99) |  | 0.67 (0.57 to 0.78)* |  | 0.78 (0.58 to 1.04) |  | 0.69 (0.56 to 0.84)* |
| Groundwater using (Abnormal versus Normal) | 1.83 (1.26 to 2.67)* |  | 1.22 (0.90 to 1.64) |  | 1.16 (0.61 to 2.19) |  | 1.61 (0.99 to 2.64) |

GroupⅠ: participants without DM and HTN; GroupⅡ: participants with HTN without DM; GroupⅢ: participants with DM without HTN; GroupⅣ: participants with DM and HTN. HTN: hypertension; DM: diabetes mellitus; HB: hepatitis B; HC: hepatitis C. OR: odds ratio for variation groups compared with reference groups on CKD; 95% CI: 95% confidence interval; ref.: reference groups.

***p*_interaction_**: p value of interaction between each factor and groups on CKD. *: significance after Bonferroni adjustment: p value < 0.05/14 = 0.0036. £: the ORs in four groups had significant heterogeneity.

TABLE S2 Effect of risk factors on CKD after adjusted demographic characteristics, history of disease and lifestyle.

|  | GroupⅠ |  | GroupⅡ |  | GroupⅢ |  | GroupⅣ |
| --- | --- | --- | --- | --- | --- | --- | --- |
|  | OR (95% CI) |  | OR (95% CI) |  | OR (95% CI) |  | OR (95% CI) |
| Gender (Male versus Female) | 1.61 (1.36 to 1.91)* |  | 1.62 (1.36 to 1.93)* |  | 1.72 (1.22 to 2.42)* |  | 1.51 (1.19 to 1.92)* |
| Age (per 10 years) | 1.12 (1.07 to 1.18)* |  | 1.02 (0.96 to 1.09) |  | 1.28 (1.12 to 1.46)* |  | 1.19 (1.09 to 1.31)* |
| Obesity (Abnormal versus Normal) | 1.15 (0.83 to 1.58) |  | 0.83 (0.63 to 1.10) |  | 1.20 (0.72 to 1.99) |  | 1.27 (0.92 to 1.77) |
| Income (Median versus Low) | 0.52 (0.44 to 0.63)* |  | 0.77 (0.63 to 0.93) |  | 0.45 (0.31 to 0.64)* |  | 0.59 (0.45 to 0.76)* |
| (High versus Low) | 0.42 (0.34 to 0.52)* |  | 0.65 (0.52 to 0.82)* |  | 0.54 (0.35 to 0.84) |  | 0.65 (0.47 to 0.91) |
| HB (Abnormal versus Normal) | 1.29 (0.98 to 1.70) |  | 1.43 (1.01 to 2.02) |  | 0.87 (0.42 to 1.83) |  | 1.00 (0.60 to 1.68) |
| HC (Abnormal versus Normal) | 1.54 (0.79 to 2.99) |  | 1.12 (0.63 to 1.99) |  | 0.80 (0.26 to 2.43) |  | 1.26 (0.57 to 2.79) |
| Hyperuricaemia (Abnormal versus Normal) | 3.72 (2.72 to 5.10)* |  | 3.69 (2.95 to 4.63)* |  | 2.80 (1.63 to 4.80)* |  | 3.76 (2.68 to 5.26) |
| Anaemia (Abnormal versus Normal) £ | 2.69 (2.03 to 3.56)* |  | 7.93 (5.46 to 11.52)* |  | 3.93 (1.89 to 8.18)* |  | 5.51 (3.49 to 8.70) |
| Hyperlipidaemia (Abnormal versus Normal) £ | 1.75 (1.40 to 2.19)* |  | 0.99 (0.82 to 1.19) |  | 1.48 (1.04 to 2.09) |  | 1.09 (0.87 to 1.36) |
| Smoking status (Abnormal versus Normal) | 1.33 (1.04 to 1.69) |  | 1.04 (0.83 to 1.31) |  | 1.20 (0.79 to 1.82) |  | 1.16 (0.85 to 1.58) |
| Alcohol intake (Abnormal versus Normal) | 0.82 (0.61 to 1.09) |  | 0.79 (0.61 to 1.02) |  | 0.73 (0.46 to 1.16) |  | 0.99 (0.70 to 1.39) |
| Betel nut chewing (Abnormal versus Normal) | 1.27 (0.74 to 2.17) |  | 0.97 (0.60 to 1.56) |  | 0.97 (0.51 to 1.85) |  | 1.22 (0.74 to 2.02) |
| Exercise habits (Abnormal versus Normal) | 0.75 (0.64 to 0.89)* |  | 0.69 (0.58 to 0.81)* |  | 0.70 (0.51 to 0.97) |  | 0.69 (0.55 to 0.86) |
| Groundwater using (Abnormal versus Normal) | 1.42 (0.94 to 2.14) |  | 1.11 (0.79 to 1.55) |  | 1.19 (0.59 to 2.38) |  | 1.70 (1.02 to 2.86) |

GroupⅠ: participants without DM and HTN; GroupⅡ: participants with HTN without DM; GroupⅢ: participants with DM without HTN; GroupⅣ: participants with DM and HTN. HTN: hypertension; DM: diabetes mellitus; HB: hepatitis B; HC: hepatitis C. OR: odds ratio for variation groups compared with reference groups on CKD; 95% CI: 95% confidence interval; ref: reference groups. ***p*_interaction_**: p value of interaction between each factor and groups on CKD. *: significance after Bonferroni adjustment: p value < 0.05/14 = 0.0036. £: the ORs in four groups had significant heterogeneity.
